# Research on Circuit Breaker Operating Mechanism Fault Diagnosis Method Combining Global-Local Feature Extraction and KELM

**DOI:** 10.3390/s24010124

**Published:** 2023-12-26

**Authors:** Qinzhe Liu, Xiaolong Wang, Zhaojing Guo, Jian Li, Wei Xu, Xiaowen Dai, Chenlei Liu, Tong Zhao

**Affiliations:** 1 School of Electrical Engineering, Shangdong University, Jinan 250100, China; qinzhe111@163.com (Q.L.); dxw@mail.sdu.edu.cn (X.D.); liu_cl@sdu.edu.cn (C.L.); zhaotong@sdu.edu.cn (T.Z.); 2 Taikai Automation Co., Ltd., Taian 271000, China; uvw2008@126.com (Z.G.); 13625382576@163.com (W.X.); 3 Taikai Disconnector Co., Ltd., Taian 271000, China; tkglqyfzb@163.com

**Keywords:** breaker, vibration signal, S-transform, gray level co-occurrence matrix, extreme learning machine

## Abstract

In response to the lack of generality in feature extraction using modal decomposition methods and the susceptibility of diagnostic performance to parameter selection in traditional mechanical fault diagnosis of high-voltage circuit breaker operating mechanisms, this paper proposes a Global-Local feature extraction method based on Generalized S-Transform (S-Translate) combined with Gray Level Co-Occurrence Matrix (GLCM) and complemented by Maximum Relevance and Minimum Redundancy (mRMR) feature selection. The GL (Global-Local)-mRMR-KELM fault diagnosis model is proposed, which employs the Kernel Extreme Learning Machine (KELM). In this model, the original time-frequency domain features and the time-frequency features of the Generalized S-Transform matrix of vibration signals under different states of the circuit breaker are first extracted as global features. Then, the GLCM is obtained to extract texture features as local features. Finally, the mRMR and KELM are comprehensively applied to perform feature selection and classification on the dataset, thereby accomplishing the fault diagnosis of the circuit breaker’s operating mechanism. In this study, the 72.5 kV SF_6_ circuit breaker operating mechanism is taken as the research object, and three types of mechanical faults are simulated to obtain a vibration signal. Experimental results verify the effectiveness of the proposed GL-mRMR-KELM model, achieving a diagnostic accuracy of 96%. This research provides a feasible approach for the fault diagnosis of circuit breaker operating mechanisms.

## 1. Introduction

High-voltage circuit breakers are crucial equipment in power grids for interrupting electrical circuits. However, during their long-term operation, they may experience faults, with a majority of them being mechanical failures in the operating mechanism. If these faults can be detected early, treatment can be taken in advance to avoid serious faults. Therefore, early diagnosis and fault prediction of mechanical failures in circuit breaker operating mechanisms are of significant importance for the safe and stable operation of power systems.

Research on the diagnosis of mechanical faults in circuit breaker operating mechanisms, both domestically and internationally, has focused on vibration signal feature extraction methods and fault classification models. During the operation process of high-voltage circuit breakers, the components are interlinked and mutually influenced, resulting in vibration signals containing a large amount of redundant information. Extracting these features poses challenges such as feature complexity and high dimensionality, which significantly increases the requirements for feature extraction algorithms. Inadequate feature extraction may result in insufficient representation, while excessive feature dimensionality could lead to redundant representation, both of which can affect diagnostic performance [1,2].

Currently, the main methods for feature extraction from circuit breaker vibration signals to address these problems include short-time energy analysis, wavelet packet analysis, phase space reconstruction, empirical mode decomposition (EMD), and variational mode decomposition (VMD) [3,4,5,6,7,8]. Among these, using wavelet or modal analysis to decompose the signals for feature extraction is the predominant approach. However, due to the differences in mechanical structures of different equipment, these methods often require experimental selection of feature extraction parameters when applied to other types of devices.

The models used as classifiers in circuit breaker operating mechanism condition diagnosis mainly include support vector machines (SVM), clustering, random forests, neural networks, etc. [9,10,11,12,13,14,15,16,17]. While SVM is the most widely used model, its drawback is that training is relatively difficult and prone to local optima in small-sample situations. Obtaining high-voltage circuit breaker operating mechanism fault state data is challenging, making it difficult to collect a large number of samples. The application of convolutional neural networks (CNN), residual networks (ResNet), and other typical neural networks is limited by the issue of effective data quantity [18,19,20]. The high training and data costs also make it difficult to transfer their applications between different types of equipment. Further research is needed to explore their applicability in circuit breaker operating mechanism condition diagnosis.

To address the aforementioned issues, a related study [21] used the generalized S-transform to obtain the time-frequency spectrum of current signals during series arc faults and extracted energy and root mean square as features for fault classification. Another study [22] utilized the gray-level co-occurrence matrix (GLCM) to extract texture features from the time-frequency spectrum of load tap changer switches, improving the accuracy and fault detection rate in early fault identification. In literature [23], local binary pattern (LBP) is used to convert the bearing vibration signal into a grayscale image and successfully extract its fault characteristics, finally reaching an accuracy of 96%. In this paper, we integrate the above methods and process global and local features separately during the feature extraction stage. Firstly, we extract the original time-frequency domain and time-frequency features of the generalized S-transform matrix from the vibration signals of circuit breaker operating mechanisms under different conditions, considering them as global features. At the same time, we extract the local features of the vibration signals separately using the 1D-LBP combined with GLCM’s texture feature extraction method. This approach reduces the reliance on detailed features obtained through modal decomposition and enhances the generality of the feature extraction method. The features are further selected using the maximum relevance and minimum redundancy (mRMR) method, and the resulting feature set is referred to as the global-local feature extraction method GL (Global-Local)-mRMR in the following text.

In addition, this paper introduces the kernel extreme learning machine (KELM) as a classifier, which has a lower training difficulty compared to SVM, to improve the performance of the model with small-sample data. Finally, by combining GL-mRMR and KELM for feature extraction and classification, the state diagnosis of circuit breaker operating mechanisms is achieved. The effectiveness of the proposed method is demonstrated by analyzing the test results of a 72.5 kV SF_6_ circuit breaker operating mechanism under different operating states.

## 2. Vibration Signal Feature Extraction

### 2.1. Analysis of Circuit Breaker Vibration Signals

**Figure 1:**
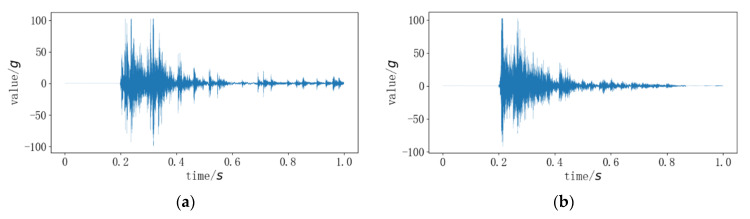
Vibration data when the circuit breaker is normally opened and closed; (**a**) open signal; (**b**) close signal.

During the operation of the circuit breaker operating mechanism, the vibration signals undergo significant changes in a short period, as shown in Figure 1.

During the startup phase, the vibration signal undergoes frequency and amplitude variations, with a sudden increase in energy signals within a short period. In the acceleration phase, high-frequency signals reappear, reaching the second peak. In the deceleration phase, the frequency and amplitude decrease. If abnormal vibrations occur at this stage, it may be due to damaged, jammed, or loose components of the mechanism.

The diagnosis of the circuit breaker operating mechanism requires the analysis of features throughout the entire process. In this section, we propose a multi-level global-local feature extraction method (GL-mRMR) for vibration signals under abnormal states of the circuit breaker operating mechanism. The features are divided into global features and local features and extracted independently. Then, using the maximum relevance and minimum redundancy approach, the features are selected to obtain the feature set for classification.

### 2.2. Global Feature Extraction

For the selection of global features, the collected vibration data from the circuit breaker is transformed into a multi-domain dataset, and then the feature extraction method is performed on time-domain, frequency-domain, and time-frequency domain data. In the time domain, features such as mean, standard deviation (Std), root mean square (RMS), skewness, kurtosis, peak-to-peak value, impulse factor, and shape factor can be extracted. Frequency-domain features include center frequency (CF), mean square frequency (MSF), root mean square frequency (RMSF), frequency variance (VF), frequency standard deviation, median frequency amplitude, maximum frequency value, and median frequency value [24]. Time-frequency domain features can be extracted using the generalized S-transform. The S-transform combines the short-time Fourier transform and wavelet transform, overcoming the issue of fixed window size in the short-time Fourier transform and possessing the multi-resolution analysis capability of the wavelet transform [25].

By using a hyperbolic function to improve the window function, the generalized S-transform achieves higher frequency resolution for high-frequency components and higher time resolution for low-frequency components. This provides better processing results for non-periodic and non-stationary signals such as the vibration signals of circuit breaker operating mechanisms. The calculation formula is as follows:

(1) $$\begin{array}{l} S(\tau ,f)=\int_{-\infty }^{+\infty }h(t)\left[\frac{2\left|f\right|}{\sqrt{2\pi }(\gamma_{f}+\gamma_{b})}\right.\left.e^{{\left[-\frac{f^{2}[V(\tau -t,\{\gamma_{f},\gamma_{b}\lambda^{2}\})]}{2}\right]}^{2}}e^{-2\pi jft}\right]\\ V(\tau -t,\{\gamma_{f},\gamma_{b}\lambda^{2}\})=\frac{\gamma_{f}+\gamma_{b}}{2\gamma_{f}\gamma_{b}}(\tau -t-\xi )+\frac{\gamma_{f}-\gamma_{b}}{2\gamma_{f}\gamma_{b}}\sqrt{{\left(\tau -t-\xi \right)}^{2}+\lambda^{2}} \end{array},$$

In Equation (1), where *h(t)* represents the vibration signal to be processed, *f* is the signal frequency, *τ* is the signal offset, *γ_f_* and *γ_b_* are the forward and backward tapering parameters, and *λ* is the positive curvature parameter. These three parameters control the shape of the window, *ξ* is the translation factor used to ensure that the peak of the signal appears at *τ = t* [26].

The calculation formula of *ξ* is:

(2) $$\xi =\sqrt{\frac{{\left(\gamma_{f}-\gamma_{b}\right)}^{2}\lambda^{2}}{4\gamma_{f}\gamma_{b}}},$$

**Figure 2:**
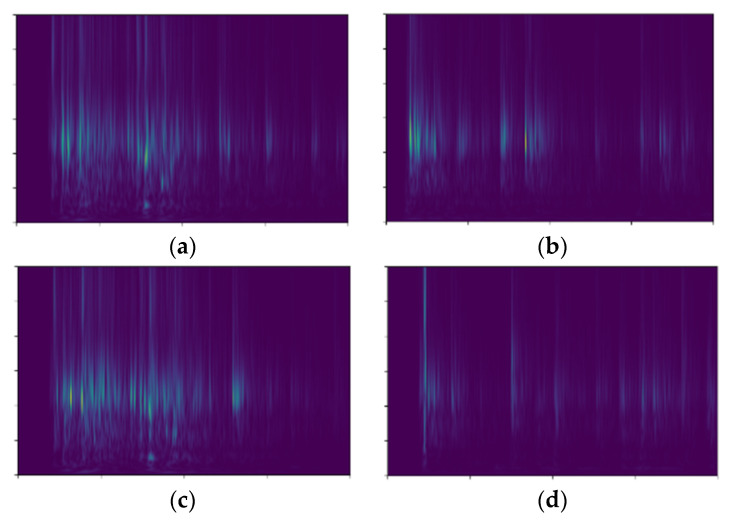
Extraction of Time-Frequency Spectrum of Closing Signal using Generalized S-Transform; (**a**) Normal closing operation; (**b**) Loose closing electromagnet; (**c**) Abnormal output force of tripping spring; (**d**) Loose fastening bolt of oil dashpot.

The parameters *γ_f_*, *γ_b_* and *λ* in Equation (2) are determined based on a population optimization method, as described in references [27,28]. The selected parameter values are *γ_f_ = 0.5, γ_b_ = 1.32, λ = 0.002*. With these parameter values, the feature extraction results are shown in Figure 2.

For the feature matrix obtained through the generalized S-transform, the following features are extracted: H1: Sum of column standard deviations, H2: Sum of row standard deviations, H3: Maximum value, H4: Sum of column variances, H5: Sum of variances along the main diagonal, H6: Sum of column kurtosis, and H7: Sum of row skewness. These 7 features can be used to compare the frequency, energy distribution, and spectral morphology of different datasets [29].

### 2.3. Local Feature Extraction

The extraction of local features is carried out in three steps. First, the one-dimensional Local Binary Patterns (1D-LBP) method is used to obtain a one-dimensional signal that reflects local variations. Second, the gray-level co-occurrence matrix (GLCM) is extracted from this signal. Finally, matrix features are extracted from the GLCM as local features.

The formula for processing one-dimensional data using LBP is as follows:

(3) LBP(x[i])=∑r=0P2−1{S[x[i+r−P2]−x[i]]×2r+s[x[i+r+1]−x[i]]×2r+P2},

The signal length is set to N, and *P* + 1 points centered around a certain sampling point *x[i]* are thresholded. The range of the processed signal is [*P/2, N-P/2*]. The differences between the sample points are then transformed into a P-bit binary code using the Sign function *S[x]*. Additionally, a binomial weight is applied to convert the binary code into an LBP code ranging from [*0, 2P*] [30]. The formulation of *S[x]* is as follows:

(4) $$S[x]=\left\{\begin{array}{c} 1,x\geq 0\\ 0,x<0 \end{array}\right.,$$

The vibration of circuit breakers exhibits strong transience, and even minor faults can cause abnormal fluctuations in local signals. By using LBP, we can obtain a description of the data differences in the local samples. Furthermore, by using GLCM on the LBP signals, we can amplify the local data features.

GLCM creates a gray-level image based on the distance (D) between pixels, the pixel angle (θ), and the gray levels (0–64), and extracts features from the image [31]. GLCM rescales the one-dimensional grayscale data based on the determined number of gray levels. Then, it assigns the sum of adjacent pixels with the specified gray level at the specified angle and distance in the rescaled data to the corresponding GLCM matrix. Since GLCM processes one-dimensional grayscale data in this case, there is no angular information involved.

**Figure 3:**
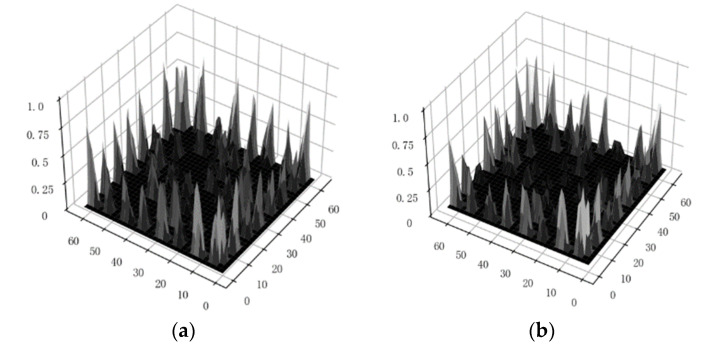

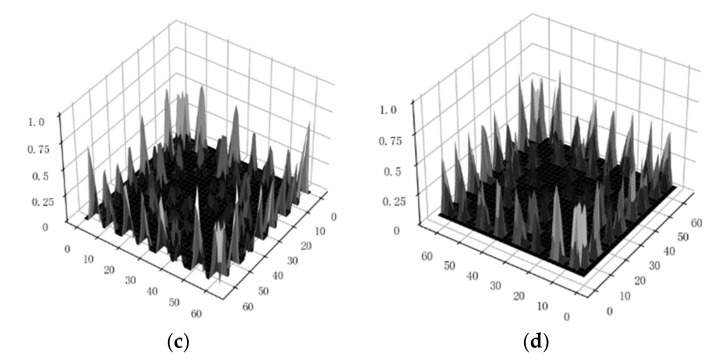
Demonstration of Gray-Level Co-occurrence Matrix for Different Types of Data; (**a**) Normal closing operation; (**b**) Loose closing electromagnet; (**c**) Abnormal output force of tripping spring; (**d**) Loose fastening bolt of oil dashpot.

Figure 3 shows the image representation of the gray-level co-occurrence matrix computed from two sets of data with different fault types. The LBP parameter P is set to 6, and the GLCM has 64 gray levels.

In the study of gray-level co-occurrence matrix (GLCM) features, the key points of interest are angular second moment (energy), entropy, correlation, contrast, and homogeneity [22,32]. The formulas for calculating these features are as follows:

(5) $$\begin{array}{l} \mathrm{Energy}=\sum_{i,j}p{\left(i,j\right)}^{2}\\ \mathrm{Entropy}=\sum_{i,j}-\ln(p(i,j))p(i,j)\\ \mathrm{Coorrelation}=\sum_{i,j}\frac{\left(i-\mu_{i})(j-\mu_{j})p(i,j\right)}{\sigma_{i}\sigma_{j}}\\ \mathrm{Contrast}=\sum_{i,j}{\left|i-j\right|}^{2}p(i,j)\\ \mathrm{Homogeneity}=\sum_{i,j}\frac{p(i,j)}{1+\left|i-j\right|} \end{array},$$

In the equations, *p(i, j)* represents the values of the gray-level co-occurrence matrix (GLCM), *μ* represents the corresponding values of the gray levels, and σ represents the standard deviation of the GLCM matrix for the gray levels.

### 2.4. Feature Selection

The mRMR method uses mutual information to measure the correlation, and the formula for calculating mutual information is as follows:

(6) $$I(x,y)=\iint p(x,y)\log\frac{p(x,y)}{p(x)p(y)}dxdy,$$

where *x* and *y* represent two variables, *p(x, y)* represents the joint probability density function, and *p(x)* and *p(y*) are the probability densities of x and y, respectively. *I(x, y)* represents the mutual information between x and y [33].

By considering the circuit breaker fault type as c and the original feature vector as xi, we can calculate the mutual information *I(x_i_, c)* between *x_i_* and *c*. This allows us to obtain the maximum correlation between features and the class, as well as the minimum redundancy between features. The formula for calculating this is as follows:

(7) $$\begin{array}{l} \max\;D(S;c),D=\frac{1}{\left|S\right|}\sum_{x_{i}\in S}I(x_{i},c)\\ \min\;R(S),R=\frac{1}{{\left|S\right|}^{2}}\sum_{x_{i},x_{j}\in S}I(x_{i},x_{j}) \end{array},$$

where *S* is the feature set and *|S|* is the number of features. The maximum correlation criterion helps identify features in the feature set that have high mutual information with the class. When there are dependencies among features or when there is redundancy in the features due to high dimensionality, the maximum correlation criterion may fail, and minimum redundancy criteria are needed to select features.

The result of mRMR is the difference between *D* and *R* in Equation (7), that is:

(8) max Φ(D,R),Φ=D−R,

Ultimately, mRMR is applied to feature selection tasks by selecting the top m features based on the magnitude of Φ.

## 3. Proposed Diagnostic Method

### 3.1. ELM Mechanism

In the ELM network, all hidden layer node weights and biases are randomly generated, and the output weights are obtained analytically using the batch least squares method [34]. In this paper, after feature extraction, the sample features are classified using ELM.

Let *ω* and *β* be the connection weights between the hidden layer, the input layer, and the output layer, respectively. The bias of the hidden layer is denoted as *b*, and the activation function is represented as *f(x)*. The ELM model can be expressed as:

(9) $$y_{j}=\sum_{i=1}^{L}\beta_{i}f(\omega_{i}\cdot x_{j}+b_{i}),j=1,2,\ldots ,N,$$

where *y* is the network output, *H* is the hidden layer output, and *T* is the network output. The matrix form of ELM can be represented as:

(10) $$H\beta =T^{T},$$

By solving the least squares, the output weights *β* can be obtained, and its solution can be expressed as:

(11) $$\beta =H^{T}{\left(HH^{T}\right)}^{-1}T,$$

To avoid matrix inversion, the generalized inverse matrix *H+* of *H* is introduced, simplifying the calculation formula to:

(12) $$\hat{\beta }=H^{+}T,$$

In ELM, the kernel function serves as the activation function for achieving nonlinear mapping from inputs to outputs, making it the key to the network’s nonlinear classification capability [35]. The selection of the kernel function needs to satisfy Mercer’s theorem. In this paper, a hybrid kernel function combining the Gaussian kernel function and the sigmoid function is proposed, and its formula is as follows:

(13) $$K(x_{i},x_{j})=\lambda \tanh[v(x_{i},x_{j})+a]+(1-\lambda )\exp\left\{-\frac{\left\|x_{i}-x_{j}\right\|}{2\sigma^{2}}\right\},$$

The regularization penalty term is introduced in the kernel function to enhance its level of nonlinearity, allowing it to have better mapping capability for non-periodic and drastic changes in data and thereby improving the generality of the classification model.

### 3.2. GL-mRMR-KELM Diagnosis Model

The choice of classifier has a significant impact on improving the fault classification performance. Since obtaining circuit breaker fault data is difficult, it is often preferred to have better performance in small sample scenarios. In such cases, ELM often outperforms Support Vector Machines (SVM). This is because ELM’s parameters are randomly generated, resulting in fewer training parameters compared to single-hidden-layer neural networks. ELM does not require backpropagation to compute gradients, resulting in faster training speeds and the ability to handle high-dimensional data.

In contrast, SVM may suffer from overfitting or underfitting, requiring more parameter tuning and data preprocessing, and longer training times. KELM improves the nonlinear classification capability based on ELM.

**Figure 4:**
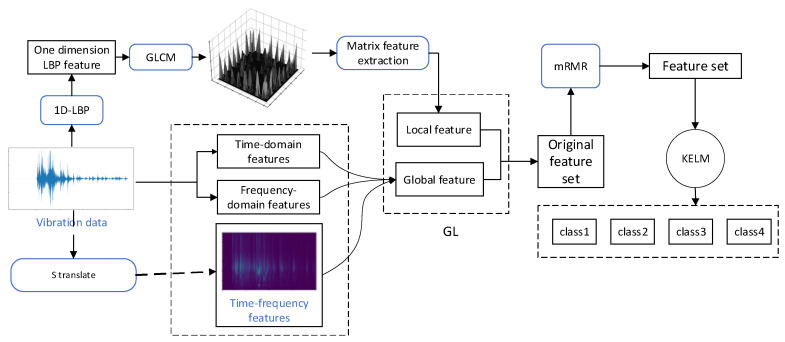
Structure of GL-mRMR-KELM.

In this section, a GL-mRMR-KELM model is proposed for classifying circuit breaker vibration data, as shown in Figure 4. The GL-mRMR feature extraction is combined with the KELM classifier.

As shown in Figure 4, the formula for obtaining feature *F* is as follows

(14) $$F(x)=F_{L}(x)+F_{G}(x),$$

In Formula (14), *F_L_* is a local feature, which is obtained from the texture features obtained from the gray-level co-occurrence matrix. *F_G_* is a global feature, which is composed of time-frequency domain features obtained by S transformation, original time-domain features, and frequency-domain features.

Global features and local features are combined as the original feature dataset. Then, mRMR is used to eliminate redundant features and those with weak correlation to the fault information from the merged features, resulting in the final dataset. The features are then input into the KELM classifier to obtain the classification results.

## 4. Experiment

### 4.1. Dataset Processing

**Figure 5:**
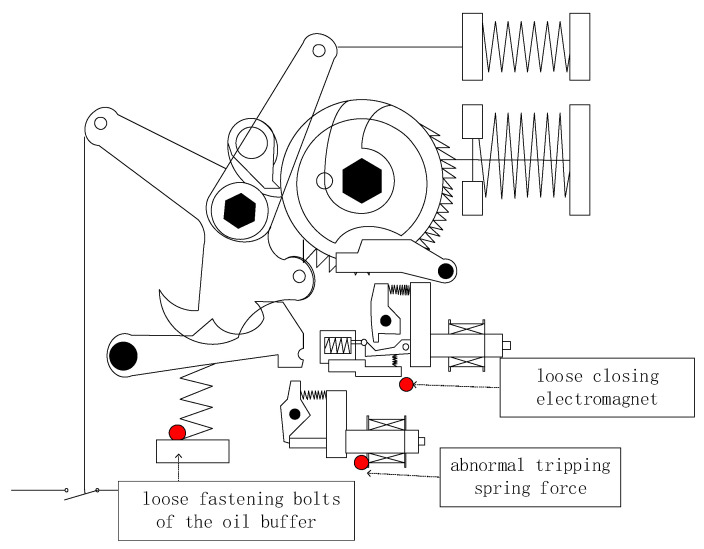
Fault Point Setting for Circuit Breaker Operating Mechanism.

The experimental equipment used is a high-voltage AC SF_6_ circuit breaker, model LW30-72.5. The sensor selected is YD-35D, and the vibration signal acquisition card is NI-9231. The sampling frequency is set to 50 kHz, and a period of redundancy is reserved for the sampling time, which is set to 1 s. The experiments include four states: normal operation, loose closing electromagnet, abnormal output of opening spring, and loose fastening bolt of oil buffer. We simulated three types of faults at different positions of the circuit breaker as shown in Figure 5. The reason why these three positions were selected is that firstly they are key structural points, secondly, faults with a higher probability of occurrence were selected, and thirdly, they are easier to simulate. These states are achieved through the circuit breaker opening and closing experiments. In the above three states with abnormal changes in the operating mechanism, the circuit breaker vibration signals exhibit corresponding changes.

**Figure 6:**
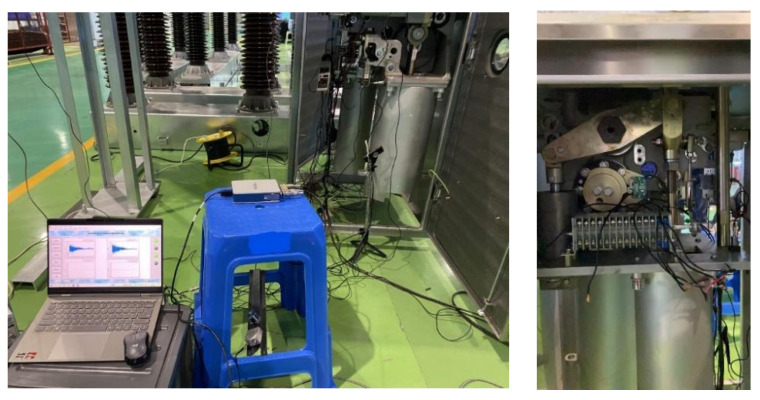
Experimental Setup for Signal Acquisition.

In the four states of the circuit breaker operating mechanism, 60 sets of opening and closing experiments were conducted for each state, resulting in a total of 240 sets of vibration data. The experimental setup for signal acquisition is shown in Figure 6.

**Figure 7:**
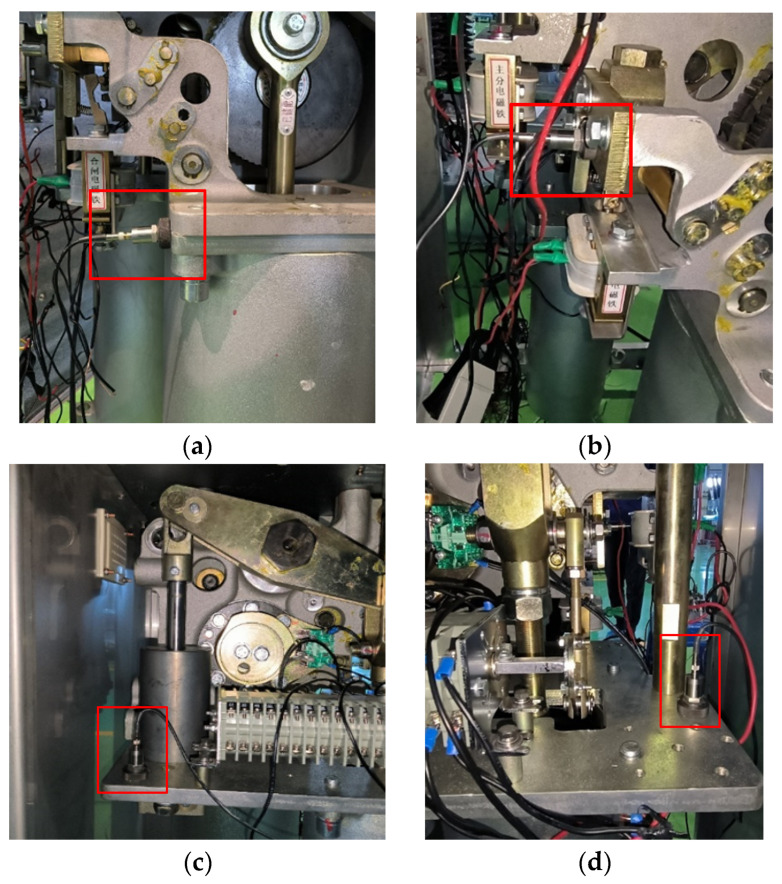
Installation positions of the four vibration sensors; (**a**) Side view of closing bushing top plate; (**b**) Connection plate of closing electromagnet; (**c**) Left top plate of opening bushing (**d**) Right top plate of opening bushing.

Considering the differences in vibration at different measurement points, four fixed measurement points were selected for data acquisition during the field experiment. To ensure the practical application and promotion of the diagnostic model, the main criteria for selecting measurement points are ease of installation and no impact on circuit breaker operation, such as the opening and closing bushing and the vicinity of the closing electromagnet. The positions are shown in Figure 7. Four vibration sensors were used to capture the vibration signals from these positions, which not only retained sufficient vibration information but also avoided redundancy.

### 4.2. Feature Extraction of Circuit Breaker Vibration Signals

By using a combination of global features and local texture features, a vibration sensor collects data and obtains a 28-dimensional feature vector before feature selection. The feature distribution consists of 8 features in the time domain, 8 features in the frequency domain, 7 features in the time-frequency domain, and 5 texture features. In the experiment, vibration signals were simultaneously collected from 4 sensors, resulting in 112 initial features. These features were then reduced using mRMR. In this paper, we tested the classification accuracy of the GL-mRMR-KELM model with varying numbers of retained features. Separate models were trained for the opening and closing states.

In the model training phase, we used data enhancement to expand the original dataset. The operations we performed included three simulations: random translation of data, random noise addition, and data interpolation. Through these methods, the data was expanded three times. In subsequent experiments, we also maintained the expanded data. The data was divided into a 60% training set and a 40% testing set for cross-validation. The diagnostic accuracy was calculated as the average of the opening and closing accuracies. The final result was obtained as the average of 20 accuracy tests.

**Figure 8:**
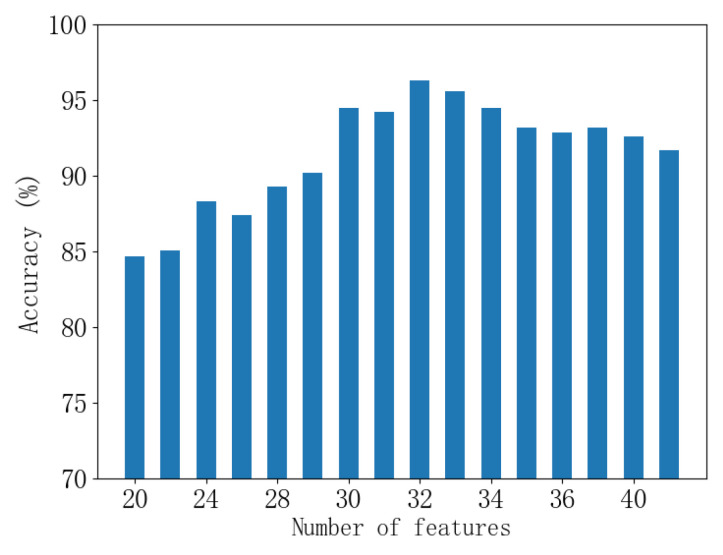
Effect of Feature Selection Parameters on Classification Performance.

As shown in Figure 8, it can be observed that the training performance decreases after retaining more than 32 features, indicating overfitting of the data. Therefore, feature selection is necessary for accurate classification of the circuit breaker. In the proposed diagnostic scheme for the circuit breaker operating mechanism vibration data, the mRMR parameter for feature retention was set to 32.

**Figure 9:**
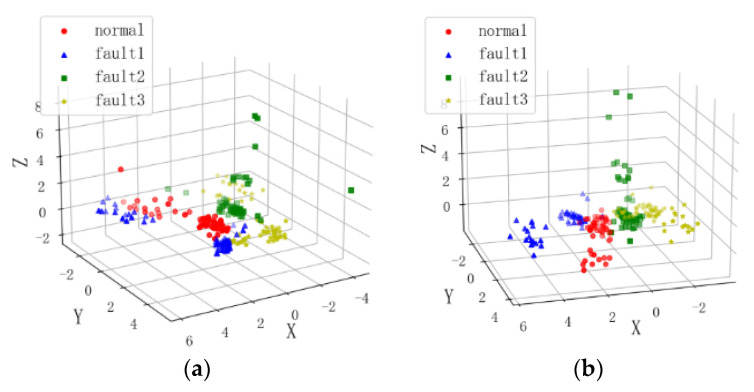
Comparison of Feature Distribution Before and After Feature Selection using mRMR; (**a**) Original Features; (**b**) Dimensionality Reduced Features.

Before and after feature selection using mRMR, the three-dimensional feature space distributions were plotted as shown in Figure 9. It can be observed that the original feature distribution is not clear, with outliers and dispersed samples. After feature selection, the feature distribution becomes more concentrated, with clear differentiation between categories. This demonstrates the effectiveness of the feature selection algorithm in extracting informative features.

### 4.3. Classification of Circuit Breaker Operating Mechanism Fault States

**Figure 10:**
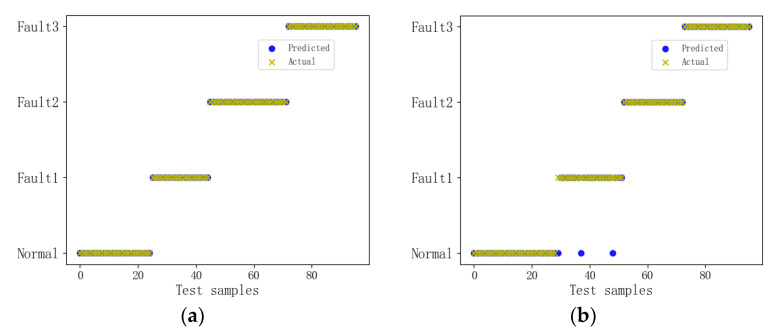
Classification Performance Demonstration; (**a**) open signal; (**b**) close signal.

After selecting the parameters, statistical analysis was performed on the four different types of data, and the classification results of the GL-mRMR-KELM model were plotted as shown in Figure 10. The testing set consisted of 40% of the dataset, totaling 96 samples.

As shown in Figure 10, through multiple experiments, it was found that the model has a higher diagnostic accuracy for the opening state compared to the closing state. The diagnostic accuracy for the opening state is close to 100%, which meets the diagnostic requirements in practical applications.

To verify the improvement in accuracy achieved by the local features, a G-mRMR-KELM model without extracting local features was used as a control. The same number of features was retained, and repeated experiments were conducted, with the average accuracy recorded. In addition, to compare the accuracy performance of the KELM classifier with other classifiers in the model, SVM and ELM were used as the classifiers in the same feature set for testing, and the accuracy was compared.

The final experimental results are shown in Table 1. Each experiment was repeated 20 times, and the average accuracy was recorded.

According to Table 1, it can be observed that the accuracy of the model classification significantly decreases when feature selection is not used. Furthermore, the performance of the model using KELM outperforms the traditional SVM and ELM models in various diagnostic categories.

**Figure 11:**
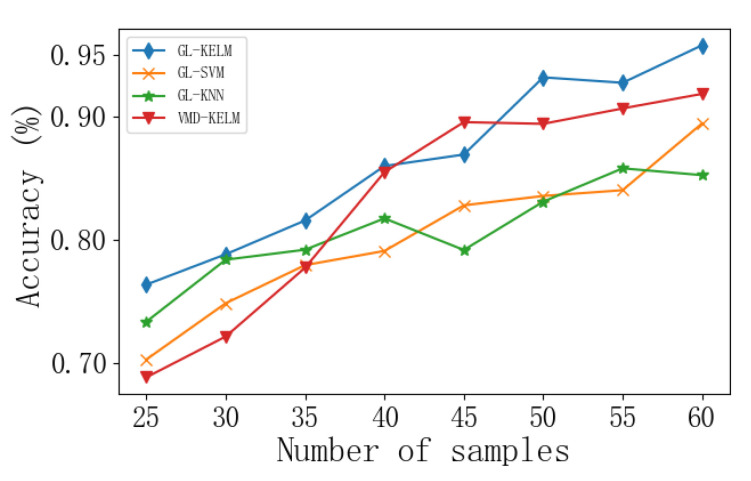
Curve of Changes in Diagnostic Accuracy of Different Classifiers with the Variation of Training Set Sample Size.

In addition, to verify the diagnostic effectiveness of the GL-mRMR-KELM model compared to the SVM and ELM models under different sample sizes, the diagnostic accuracy of the three classifiers was recorded during the experiment as the training set data varied from 80 to 144, as shown in Figure 11.

The results indicate that the proposed GL-mRMR-KELM model maintains a high level of performance even in small sample situations. Overall, the classification performance of the KELM model using the global-local feature extraction proposed in this paper outperforms SVM and ELM classifiers.

**Figure 12:**
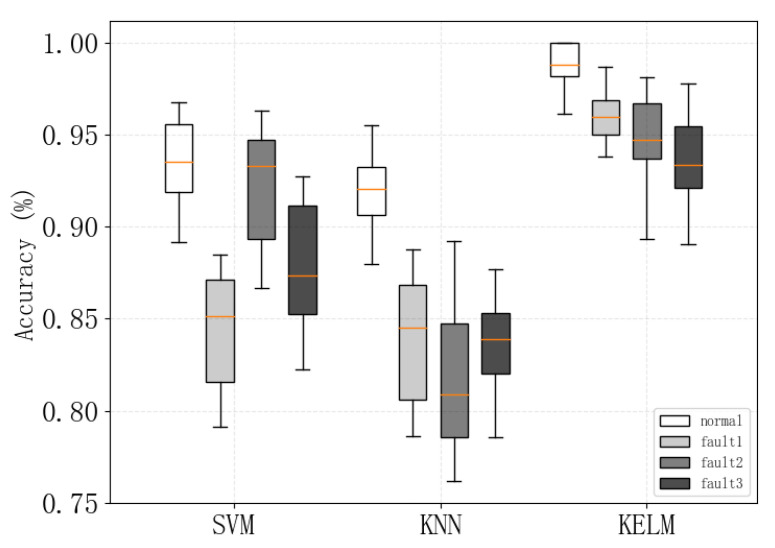
Display of Range of Diagnostic Accuracy for Different Classifiers.

In the multiple experiments conducted, the range of model accuracy was recorded when using the three different classifiers, as shown in Figure 12. It can be observed that the GL-mRMR-KELM model proposed in this paper achieves high diagnostic accuracy for various faults, and the use of KELM as the classifier also demonstrates better stability compared to other classifiers.

## 5. Conclusions

In this paper, we have investigated the fault diagnosis of the operating mechanism of SF6 circuit breakers based on vibration signals. Addressing the challenges of low generality and low accuracy under small sample conditions in current operating mechanism state diagnosis algorithms, we proposed a GL-mRMR-KELM fault diagnosis model for high-voltage circuit breakers. First, we conducted fault simulation experiments on 72.5 kV circuit breakers, where we selected three common early non-destructive faults for opening and closing experiments, resulting in a small sample dataset of 240 groups. Based on this dataset, we trained and tested the model. The experimental results demonstrate the following:

For early faults in the circuit breaker mechanical mechanism, the proposed GL-mRMR-KELM model can effectively distinguish different operating conditions, achieving an average accuracy of 95.76% in classification.

We proposed a method that uses local binary patterns combined with a gray-level co-occurrence matrix to detect the local texture features of vibration signals in circuit breakers. The inclusion of local features improves the diagnostic accuracy of the training model. Compared with the feature dataset without local features, the diagnostic accuracy is improved by 12.71%. Additionally, comparisons were made between KELM and SVM, ELM as classifiers, resulting in accuracy improvements of 6.34% and 10.53%, respectively.

The use of mRMR for feature selection effectively filters out redundant features. Compared to the original feature space, the feature distribution is significantly improved.

This model provides an effective diagnostic model training solution for circuit breaker models that can be fault simulated. It can still achieve good fault diagnosis results even when the amount of data is small, and the model training method is relatively simple. No need to make too many parameter adjustments. Compared to other circuit breaker fault diagnosis models, this model is insensitive to hyperparameter selection and demonstrates high diagnostic accuracy under small sample conditions. The method proposed in this paper, combining global and local features for feature extraction, effectively addresses the balance between high-dimensional representations leading to overfitting and the inability to extract sufficient features. Further research can explore the addition of more universal features to the global features and optimize the extraction method of local features.

In practical applications, the test results of vibration signals in circuit breakers can be affected by environmental factors. However, this model has not undergone field experiments on different types of circuit breakers, thus its universality under changing circuit breaker structures cannot be guaranteed. In addition, the duration of use of the circuit breaker may cause changes in the vibration signal, thus affecting the accuracy of diagnosis. Due to the limitations of the dataset, the model can only diagnose existing fault types and cannot diagnose fault types that cannot be simulated in practice. In further research, incorporating unsupervised learning algorithms may help improve accuracy and diagnose unknown faults. Regarding the problem of unbalanced data, because the fault types selected in this article are all faults that are convenient for actual simulation, they will not be affected by this problem. Further research is needed on the diagnosis of some faults that are difficult to obtain data.

## Figures and Tables

**Table 1:** Differences in Diagnostic Accuracy of Different Diagnostic Algorithms in Specific Types of Faults.

Model	Normal	Closing Electromagnet Loose	Tripping Spring Abnormal	Oil Buffer Bolt Loose	Average
GL-mRMR-KELM	98.79%	96.06%	94.40%	93.77%	95.76%
G-mRMR-KELM	90.68%	78.95%	80.06%	82.50%	83.05%
GL-mRMR-SVM	93.25%	84.30%	92.31%	87.84%	89.42%
GL-mRMR-ELM	92.20%	83.98%	81.35%	83.39%	85.23%

## Data Availability

The data presented in this study are available on request from the corresponding author.
